# Complete Genome Sequence of the Cesium-Accumulating Bacterium Rhodococcus qingshengii CS98, Isolated from Soil in Japan

**DOI:** 10.1128/MRA.01188-20

**Published:** 2020-12-10

**Authors:** Katsuya Satoh, Shogo Ozawa, Hidenori Hayashi, Noriko Tomioka, Issay Narumi, Yutaka Oono

**Affiliations:** a Department of Radiation-Applied Biology Research, Takasaki Advanced Radiation Research Institute, Quantum Beam Science Research Directorate, National Institutes for Quantum and Radiological Science and Technology, Takasaki, Gunma, Japan; b Faculty of Engineering, Maebashi Institute of Technology, Maebashi, Gunma, Japan; c Center for Regional Environmental Research, National Institute for Environmental Studies, Tsukuba, Ibaraki, Japan; d Faculty of Life Sciences, Toyo University, Itakura, Gunma, Japan; Portland State University

## Abstract

Rhodococcus qingshengii CS98 is a bacterium isolated from soil in Japan that shows strong cesium-accumulating ability. Here, we report the complete genome sequence of *R. qingshengii* (6.7 Mb), which may provide useful genetic information supporting its bioremediation features.

## ANNOUNCEMENT

Bioremediation uses biological organisms to solve an environmental problem, drawing attention as a technology with a low environmental burden, compared with physical and chemical technologies. The cesium-accumulating bacterium Rhodococcus erythropolis CS98 was isolated from soil in Japan (1, 2) as a promising strain for bioremediation purposes. Recently, this strain was reclassified as Rhodococcus qingshengii CS98 on the basis of the results of the average nucleotide identity analysis, and its draft genome has been reported (3).

*R. qingshengii* CS98 cells that had been stored as glycerol stocks in a −80°C ultrafreezer at the National Institute for Environmental Studies, Japan, after the initial characterization (1, 2) and revived in January 2016 were grown at 30°C for 24 h in medium (0.5% tryptone, 0.1% glucose, and 0.3% yeast extract). The genomic DNA was extracted with a DNeasy PowerSoil kit (Qiagen). For long-read sequencing, genomic DNA was sheared to ∼8-kb fragments using a g-TUBE device (Covaris). A library was constructed using a ligation sequencing kit 1D (SQK-LSK108) and a native barcoding kit 1D (EXP-NBD103) and was loaded onto a GridION X5 R9.4 flow cell (FLO-MIN106; Oxford Nanopore Technologies) with default parameters. Base calling was performed using Guppy v.1.8.5 (Oxford Nanopore Technologies), adapter sequences were removed using Porechop v.0.2.3 (https://github.com/rrwick/Porechop), and low-quality data (read length of <1,000 bp and Phred quality score of <9) were filtered and trimmed using NanoFilt v.2.0.0 (4). For short-read sequencing, a library was prepared with a TruSeq DNA PCR-free kit (Illumina) and subjected to 150-bp paired-end sequencing (300 cycles) on the MiSeq system (Illumina). Adapter sequences and low-quality data (read length of <10 bp and Phred quality score of <30) were filtered and trimmed using Cutadapt v.1.11 (5). GridION and MiSeq sequencing generated total lengths of 1,389,142,401 and 259,666,780 bp, respectively (Table 1). Hybrid *de novo* assembly was performed using Unicycler v.0.4.6 (6) with default parameters, including a polishing step with Pilon v.1.2.2 (7). Unicycler automatically identified and trimmed overlapping ends. The genome of *R. qingshengii* CS98 was 6,726,107 bp in total length, with an average G+C content of 62.4% and mean coverage of 245-fold, and comprised one circular chromosome (6,240,414 bp) and one linear plasmid (485,693 bp). The circular chromosome was rotated to the default starting gene *dnaA*. The genome was automatically annotated with the DFAST pipeline (8), which predicted a total of 6,255 protein-coding sequences, 59 tRNAs, and 5 rRNA (5S, 16S, and 23S rRNA) operons. Comparative analysis using Mauve v.2.4.0 (9) showed that the genome was almost identical with the previous draft genome (3) and complemented gaps between contigs. BLAST v.2.11.0 (https://blast.ncbi.nlm.nih.gov) analysis revealed that the chromosome sequence had similarities to chromosomes of *R. qingshengii* strains djl-6-2 (GenBank accession number CP025959) and RL1 (GenBank accession number CP042917) (identities of 99.07% and 99.09% and query coverages of 96% and 95%, respectively). Furthermore, the linear plasmid possessed plasmid partitioning gene *parA*, and the majority of the sequence showed a high level of similarity (identity of 99.15% and query coverage of 30%) to that of the *Rhodococcus* sp. strain BH4 linear plasmid (GenBank accession number CP014942). The complete genome sequence of *R. qingshengii* CS98 represents a valuable resource for future comparative genomic analyses and will help elucidate the genetic basis of bioremediation features.

**TABLE 1:** Summary of long-read and short-read sequencing results

Sequencing platform and metric	Finding before filtering	Finding after filtering
GridION		
No. of bases	2,090,455,438	1,389,142,401
No. of reads	352,550	224,929
* *Read *N*_50_ (bp)	7,596	7,662
Mean read length (bp)	5,929.5	6,175.9
Median read length (bp)	5,771.0	5,994.0
MiSeq		
No. of bases	304,966,546	259,666,780
No. of reads	2,019,646	1,879,082

### Data availability.

The complete genome sequence of *R. qingshengii* CS98 was deposited in DDBJ/EMBL/GenBank under the accession numbers AP023172 and AP023173. The BioProject and BioSample numbers are PRJDB9718 and SAMD00222554, respectively. The DDBJ Sequence Read Archive (DRA)/NCBI SRA accession number is DRA010796.
